# Genetic analysis identifies the missing parchment of New Zealand’s founding document, the Treaty of Waitangi

**DOI:** 10.1371/journal.pone.0210528

**Published:** 2019-01-16

**Authors:** Lara D. Shepherd, Peter Whitehead, Anna Whitehead

**Affiliations:** 1 Museum of New Zealand Te Papa Tongarewa, Wellington, New Zealand; 2 Alexander Turnbull Library, Wellington, New Zealand; 3 Archives New Zealand Te Rua Mahara o te Kāwanatanga, Wellington, New Zealand; University of Florence, ITALY

## Abstract

Genetic analyses provide a powerful tool with which to identify the biological components of historical objects. Te Tiriti o Waitangi | The Treaty of Waitangi is New Zealand’s founding document, intended to be a partnership between the indigenous Māori and the British Crown. Here we focus on an archived piece of blank parchment that has been proposed to be the missing portion of the lower parchment of the Waitangi Sheet of the Treaty. However, its physical dimensions and characteristics are not consistent with this hypothesis. We perform genetic analyses on the parchment membranes of the Treaty, plus the blank piece of parchment. We find that all three parchments were made from ewes and that the blank parchment is highly likely to be a portion cut from the lower membrane of the Waitangi Sheet because they share identical whole mitochondrial genomes, including an unusual heteroplasmic site. We suggest that the differences in size and characteristics between the two pieces of parchment may have resulted from the Treaty’s exposure to water in the early 20^th^ century and the subsequent repair work, light exposure during exhibition or the later conservation treatments in the 1970s and 80s. The blank piece of parchment will be valuable for comparison tests to study the effects of earlier treatments and to monitor the effects of long-term display on the Treaty.

## Introduction

Documents have often been the foundation of societies since the invention of writing. Some documents, such as the Magna Carta and the United States Constitution, remain important political symbols because of their continued relevance. Technological advances now allow new insights into the origins of these priceless artifacts [1–4]. The Treaty of Waitangi, New Zealand’s founding document, is unique because it involved an entire country and a single indigenous group. It differs from other agreements made around the world during the mid-nineteenth century because it was understood at the time by Britain that Māori were ceding indigenous sovereignty and it incorporated promises regarding the protection of indigenous land, fisheries and other rights. The Treaty continues to be of significance today because of Māori claims against the Crown for historic and present-day breaches of the principles of the Treaty. The Waitangi Tribunal is a commission of inquiry created in 1975 to consider and make recommendations on Treaty claims. Its mandate was expanded in 1985 to allow claims to be lodged on matters dating back to 1840. To date over 2000 claims have been lodged with the tribunal with unofficial estimates of the settlement fund close to NZ$2 billion [5].

The Waitangi Sheet of the Treaty was first signed at the settlement of Waitangi on the 6^th^ February 1840 and more signatures were added in subsequent days to this first parchment (the upper membrane of the Waitangi Sheet). After several weeks an additional parchment Sheet (the lower membrane of the Waitangi Sheet), which is roughly half the size of the first parchment, was attached below the first sheet to provide space for additional signatures. A separate parchment sheet, known as the Herald Sheet, as well as seven paper sheets, were used for gathering additional signatures from other regions of New Zealand over several months. In total over 500 Māori chiefs signed the Treaty.

**Fig 1:**
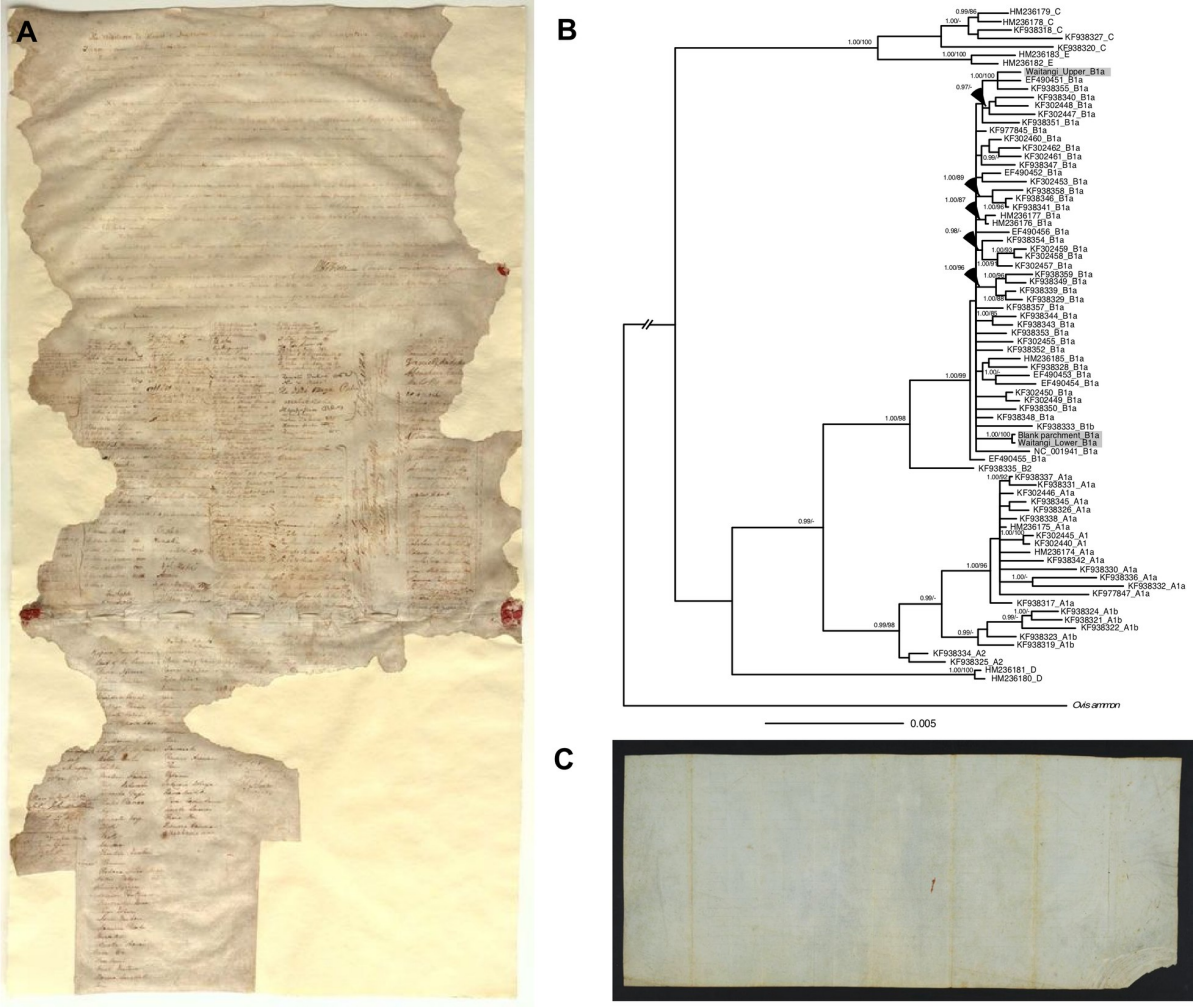
The Waitangi Sheet of the Treaty of Waitangi and phylogeny constructed from parchment mitogenomes. (A) The Waitangi Sheet of the Treaty of Waitangi showing the extensive damage to the two parchments by rats, water and subsequent repairs. (B) Bayesian consensus phylogeny constructed from *Ovis aries* mitogenomes. The haplotype grouping is given after the GenBank number. Support values are reported as follows: Bayesian posterior probability (PP)/maximum likelihood bootstrap (BS). Only values above 0.95 PP and 80% BS are shown. (C) The blank piece of parchment that we show is likely to be a portion removed from the lower membrane of the Waitangi Sheet.

Despite the historical importance of the Treaty of Waitangi it suffered from a lack of proper care during the 19^th^ and early 20^th^ centuries. It was nearly destroyed by fire in 1841. In 1908 the Treaty was discovered in the basement of Government Buildings where it had been damaged by water and the parchment sheets partially eaten by rats. Restoration work was undertaken in 1913 with the parchments lined with canvas using a paste but this caused further damage, including hardening and further staining [6] (Fig 1A).

In 1929 a blank piece of parchment in an envelope labeled “1865, Treaty of Waitangi Blank Portion of the Original Skin” [7] was donated to the Alexander Turnbull Library with the papers of WBD Mantell, who was Minister of Native Affairs for two terms in the 1860s (Fig 1C). The Dominion Archivist at the time compared the measurements of this blank parchment with the lower membrane of the Waitangi Sheet and recorded that the lead pencil rulings matched exactly and that he had no doubt that the blank parchment had been cut off the Treaty (S1 File). However, the damage that had already occurred to the lower membrane of the Waitangi Sheet by 1929 would have made it difficult to compare the rulings. Our own measurements indicate that the blank piece of parchment is now 29 mm wider than the lower membrane of the Waitangi Sheet (S1 File) and displays significantly different physical characteristics including colour, thickness and texture, casting doubt on its authenticity. Positive identification of the piece of parchment as belonging to the Treaty would increase its historical value and be invaluable for understanding the effects of past conservation treatments, long-term display and lighting on the Treaty.

Parchment has been shown to be a good source of DNA [3,4,8] although some studies have reported issues with contamination when using Sanger sequencing [9, 10]. Here we use ancient DNA analyses to determine whether the blank piece of parchment is a missing portion of the lower membrane of the Waitangi Sheet.

## Materials and methods

### Sampling and DNA extractions

The Treaty of Waitangi is currently stored at the National Library of New Zealand, where it is displayed as part of He Tohu, an exhibition of three of New Zealand’s constitutional documents. We obtained multiple samples for DNA extraction from both membranes of the Waitangi Sheet of the Treaty of Waitangi (i.e., the upper and lower membranes), the Herald Sheet, a small piece of parchment attached to the top right corner of the Herald Sheet and the blank piece of parchment suggested to be a missing section of the lower membrane of the Waitangi Sheet.

We took multiple samples using different techniques from each parchment (Table 1), including scraping material off the parchment surface with a sterile razor blade, removal of small pieces (1mm x 2mm, or less) of parchments from the verso or edge with a sterile razor blade, and the collection of eraser fragments or erdu [1, 3, 11] by rubbing the surface of the parchment with an eraser.

**Table 1:** Parchments, sampling strategies used for obtaining DNA in this study and DNA results. The primers used for 16S amplification were universal primers and for the control regions were sheep specific.

Sample	Parchment of origin	Sampling method	DNA results		
			Sanger sequencing		Illumina library prepared
			16S	Control region	
Wai5	Treaty of Waitangi, Waitangi Sheet upper membrane	Cut piece	Yes, 3 of 3 PCRs–mixed signal (sheep and cow)	Yes	Yes
Wai4	Treaty of Waitangi, Waitangi Sheet upper membrane	Cut piece	Yes (sheep)	Yes	-
Wai3	Treaty of Waitangi, Waitangi Sheet upper membrane	Eraser rubbing	n/a	n/a	-
Wai2	Treaty of Waitangi, Waitangi Sheet lower membrane	Cut piece	n/a	Yes	-
Wai1	Treaty of Waitangi, Waitangi Sheet lower membrane	Cut piece	Yes (sheep)	Yes	Yes
Blank1	Blank piece of parchment	Eraser rubbing	n/a	n/a	-
Blank2	Blank piece of parchment	Cut piece (small)	Yes (sheep)	n/a	-
Blank3	Blank piece of parchment	Cut piece	Yes (sheep)	Yes	Yes
Herald1	Treaty of Waitangi, Herald Sheet	scraping	Yes– 1 of 3 PCRs (cow)	n/a	-
Herald2	Treaty of Waitangi, Herald Sheet	Cut piece	n/a	n/a	-
Herald3	Treaty of Waitangi, Herald Sheet	Eraser rubbing	n/a	n/a	-
Herald4	Treaty of Waitangi, Herald Sheet	Eraser rubbing	n/a	n/a	-
Small	Small piece of parchment attached to the back of the Herald Sheet	Cut piece	Yes (cow)	-*	Yes

n/a = no amplification

- PCR not attempted

* DNA extract used up in library prep so not available for control region PCR

All DNA extractions and PCR set-ups were performed in a dedicated ancient DNA laboratory, physically isolated from where modern DNA and PCR products are handled. No other samples from domesticated animals had been analysed in either the ancient or modern laboratories. Potential contamination was monitored for by the use of negative extraction and PCR controls. Genomic DNA was isolated using a Qiagen DNeasy blood and tissue kit. Samples were incubated in Buffer ATL and proteinase-K was for 2 hours (erdu) or overnight (scrapings and cut pieces of parchment). Following incubation, the manufacturer’s instructions were followed, except that the final elution used 45 μl of Buffer AE and was spun through the column twice (i.e., the first elution was placed back on the column and spun through a second time).

### PCR and Sanger sequencing

Initial species identification was performed with Sanger sequencing. The universal mammal primers 16Smam1 and 16Smam2 were combined with the human blocking primer 16Smam_blkhum3 [12] to amplify a 132 base pair (bp) DNA fragment of the mitochondrial 16S gene including primers, while blocking amplification of human DNA. PCR amplifications were performed in 12 μl volumes containing 1× MyTaq buffer, 0.4 μM each of 16Smam1 and 16Smam2, 2.0 μM of 16Smam_blkhum3 and 0.04 μg human serum albumin (HSA). Thermocycling followed the protocol of Boessenkool et al. 2012 [12].

A second PCR was performed to amplify a 144 bp fragment of the mitochondrial control region including primers using the sheep-specific primers L15391 and H15534 [13]. PCR amplifications were performed in 12 μl volumes containing 1× MyTaq buffer, 0.4 μM each primer and 0.04 μg HSA. Thermocycling conditions were 94°C for 2 minutes, followed by 55 cycles of 94°C for 20 seconds, 50°C for 20 seconds and 72°C for 20 seconds, with a final extension of 72°C for 5 minutes.

PCR products were visualized by electrophoresis on 2% MS/1% LE agarose gels, then purified by digestion with 1 U shrimp alkaline phosphatase (rSAP; New England Biolabs) and 5 U exonuclease I (EXO; New England Biolabs) at 37°C for 15 minutes, followed by inactivation of the enzymes at 80°C for 15 minutes. DNA sequencing was performed by capillary separation on an ABI 3730 at the Massey Genome Service (Palmerston North, New Zealand). Sequence identities were investigated through BLAST searches of the GenBank database [14].

### Microsatellite genotyping

Seven nuclear microsatellite loci less than 130 bp in length were trialed (S1 Table). An M13 tag (TGTAAAACGACGGCCAGT) was added to the 5’ end of the forward primer of each locus. Loci were amplified individually in 10 μL PCR reactions that contained 1 μL of DNA extract, 0.02 μM forward primer, 0.8 μM reverse primer, 0.8 μM M13 primer (labelled with FAM or HEX), 1× MyTaq mix (Bioline) and 0.04 μg HSA. PCR thermocycling conditions were an initial denaturation of 94°C for 5 minutes; 35 cycles of 94°C for 20 seconds, 55°C for 20 seconds, and 72°C for 20 seconds; followed by 10 cycles of 94°C for 20 seconds, 53°C for 20 seconds, and 72°C for 20 seconds, with a final extension at 72°C for 15 minutes. Genotyping was performed on an ABI 3730 Genetic Analyzer (Applied Biosystems) at the Massey Genome Service (Massey University, Palmerston North, New Zealand). Alleles were sized using the internal size standard GeneScan 500 LIZ (Applied Biosystems) and scored using the software Geneious vers. 10.2.3 (Biomatters Ltd., Auckland, New Zealand).

### Illumina sequencing

Our initial trials with Sanger sequencing and microsatellite genotyping were inconclusive and we had several spurious sequences from our Sanger sequencing (Table 1, S1 Table). Therefore we prepared sequencing libraries for four of the samples and a negative extraction control (Table 1) using a Rubicon Thruplex DNA-seq kit, following the manufacturer’s instructions. For further sequencing we selected the sample per parchment with the highest DNA concentration as measured by a Qubit 3.0 fluorometer and the Qubit dsDNA high sensitivity (HS) assay (Invitrogen). This initial DNA concentration was also used to determine the number of library amplification cycles. The lower membrane of the Waitangi sheet and the blank piece of parchment underwent 9 PCR cycles and the remaining samples were amplified with 12 PCR cycles. Libraries were purified using Agencourt AMPure XP beads, following the manufacturer’s instructions. Sequencing was undertaken on an Illumina HiSeq at the Otago Genomics & Bioinformatics Facility, University of Otago, Dunedin using 2 x 125bp (paired-end) sequencing chemistry.

Preliminary quality control of sequencing reads was performed with FastQC [15]. Sequences were trimmed of adaptor contamination and low quality bases removed from both ends using the BBDuk vers. 37.25 plugin in Geneious vers. 10.2.3 (Biomatters Ltd, Auckland, New Zealand). Sequences shorter than 30 bp were discarded, then paired end reads were merged using BBMerge in Geneious. Merged reads were normalized and error corrected using BBNorm, with minimum depth 3 and target coverage level of 40. Duplicate reads were removed using Dedupe in Geneious.

Processed reads were mapped to RefSeq mtDNA genomes (sheep—*Ovis aries*, NC001941; cow—*Bos taurus*, NC006853; goat—*Capra hircus*, NC005044 and human–*Homo sapiens*, NC012920), using the Geneious mapper with five iterations and minimum mapping quality of 30. These species have been suggested to be the most likely source species of parchment (sheep, cow, goat) or contamination (human) [3]. Assemblies were checked manually for polymorphisms with low coverage (less than three reads). Few reads from the library constructed from the parchment Small aligned to any of the four mtDNA RefSeq genomes therefore an additional alignment was performed. Reads were mapped as described above to reference mtDNA genomes of four other species that have been used to make parchment (deer (NC007704), pig (NC000845), horse (NC001640) and donkey (NC001788)).

For the upper membrane of the Waitangi Sheet, whose mitochondrial genome had lower coverage than the other parchments, three polymorphic sites were covered by fewer than three reads. One of these sites, 16349, was only covered by a single read and has not previously been reported in the DOMETREE database [16]. This nucleotide was converted to an N in subsequent analyses. Haplogroup and polymorphisms relative to the sheep reference mtDNA genome were identified for each parchment mtDNA genome using MitoToolPy [16].

### Phylogenetic analyses

Complete mtDNA genomes from seventy-seven *Ovis aries* and one *O*. *ammon* were aligned to the three parchment mitogenomes that we were able to sequence with MAFFT v7 [17] on the CBRC-AIST server [18]. The 75/76 bp tandem repeats between nucleotide positions 15650 to 15905 were difficult to align therefore they were excluded from phylogeny reconstruction, following Lv et al. 2015 [19].

For the ML analysis, the best-fit model of sequence evolution was determined using Smart Model Selection [20] and the Akaike information criterion. The PhyML v3.0 web server [21] was run with subtree pruning-regrafting and nearest-neighbour-interchange branch swapping with ten random addition trees. Branch support was assessed with 100 pseudoreplicates.

For the Bayesian analyses, performed with MrBayes v3.2.6 [22], two concurrent analyses were run with the HKY + G model, each with four Markov chains of twenty million generations and sampling every 1000 generations. Tracer v.1.6 [23] was used to assess stationarity, with the first 20% of samples discarded as ‘‘burn-in”

### Sex determination and DNA damage analysis

Sex identification was performed by aligning reads to the sheep reference genome chromosomes X (NC019484) and 6 (NC019463). These two chromosomes are similar lengths in sheep. Therefore, for females, which have two copies of the X chromosome, similar numbers of reads would be expected to map to each of these chromosomes [24]. In contrast, the number of reads mapping to the X chromosome in males is expected to be roughly half the number that map to chromosome 6.

The haplogroup-defining positions were used to estimate the contamination rate of each sample by averaging the number of mismatches at these sites [3]. MapDamage2.0 [25] was used to analyse the damage patterns in the parchment DNA.

## Results

### Sanger sequencing results

PCR testing indicated that only the cut pieces of parchment produced reliable results. However, we collected far fewer eraser crumbs than a previous study that obtained DNA from parchment using this method [4], which is likely to have contributed to our negative results for this sampling method.

16S sequences were successfully obtained from seven of the samples (Table 1). BLAST searches showed that sequences from four samples were identical to sheep (*Ovis aries*) and another two samples had sequences that matched cow (*Bos taurus*). The 16S sequence from one sample of the Waitangi Sheet upper membrane (Upper1) exhibited a number of additive nucleotide sites, which correspond to the sites that differ between the sheep and cow sequences. The PCR amplification and sequencing of this sample was repeated three times and produced the same result. The second independent extraction of this sheet (Upper2) produced only sheep 16S sequence. The remaining samples, as well as the negative extraction and PCR controls, did not produce any amplification products.

Partial control region sequences were obtained for five samples with sheep-specific primers (Table 1). Extraction duplicates were independently amplified and sequenced for both parchments of the Waitangi Sheet and these produced consistent sequences (Table 1). The lower membrane of the Waitangi Sheet and the blank piece of parchment had identical control region sequences and the sequence of the upper membrane of the Waitangi Sheet differed from this by two substitutions.

### Microsatellite genotyping

Microsatellite genotypes were obtained for four loci but only from the blank piece of parchment (S1 Table). The alleles detected were within the size ranges expected for the loci and were not found in the negative controls.

### Illumina sequencing

For the small piece of parchment attached to the top right corner of the Herald Sheet, few reads mapped to any of the reference mitochondrial genome sequences. Therefore a source species for this parchment could not be determined, even with mapping to further reference genomes. For the other three parchments we obtained sufficient reads to reconstruct complete mitogenomes with between 6.3X and 18.5X coverage (Table 2). All three were identified as sheep (*Ovis aries*), with few reads mapping to reference mtDNA sequences from other species. This finding is consistent with legal documents of the time [26].

**Table 2:** Summary statistics of the sequenced parchment libraries and reference mapping.

Parchment	Raw number of paired reads	Number of mapped unique mtDNA reads				Coverage (X)
		sheep	cow	goat	human	
Treaty of Waitangi, Waitangi Sheet upper membrane	52 826 500	1397	8	14	2	6.3
Treaty of Waitangi, Waitangi Sheet lower membrane	109 417 948	1725	21	32	1	9.0
Blank piece of parchment	63 390 080	2971	610	169	0	18.5
Small	78 956 154	9	0	1	63	n/a

The mtDNA sequences obtained from the lower membrane of the Waitangi Sheet and the blank piece of parchment were identical and both exhibited a single heteroplasmic site at nucleotide position 6907 relative to the reference sequence: lower membrane (6 reads A, 6 reads G) and the blank piece of parchment (13 reads A, 13 reads G). In contrast the mitogenome obtained from the upper membrane differed by 28 nucleotides to that sequenced from the lower membrane of the Waitangi Sheet and the blank piece of parchment

Haplogroup and polymorphisms relative to the sheep reference mtDNA genome placed all three samples placed within mitochondrial haplogroup B, which is the most common haplogroup in Europe [27] (Table 3). Phylogenetic reconstruction showed that all three samples clustered within haplogroup B1a with strong support (1.00PP/99% BS) in the phylogenetic analyses (Fig 1B).

**Table 3:** Mitogenome haplogroups for shotgun sequenced parchment libraries from the Treaty of Waitangi, Waitangi Sheet.

Parchment	GenBank Number	Haplogroup	Polymorphisms
Treaty of Waitangi, Waitangi Sheet upper membrane	MH841968	B1a	**281C,566+G,1729+C,3543A,6615A,7500A,8264C,8651T,9375G,11668A,11710deletion,12539C,12571C,13199G,13813C,14055C,15721C,15783T,15800T,15820T,16128T,16342+C**#**,16343C**#**,16472deletion** 732G,1114C,3035C,6375T,10861A,14377G,14683T,14871C,15439C,15658G,15806,16130G,16349T#,16440C
Treaty of Waitangi, Waitangi Sheet lower membrane	MH841966	B1a2a1	**281C,566+G,1729+C,3543A,6615A,7500A,7983C,8264C,8651T,9375G,11668A,11710deletion,12539C,12571C,13199G,13813C,14055C,15721C,15800T,16128T,16342+C,16343C, 16472deletion** 6907R,7573A,8193T,11149G,11653T,12851A,13097A,13489A,15487C,15708C,15787A,15858C,16440C
Blank piece of parchment	MH841967	B1a2a1	**281C,566+G,1729+C,3543A,6615A,7500A,7983C,8264C,8651T,9375G,11668A,11710deletion,12539C,12571C,13199G,13813C,14055C,15721C,15800T,16128T,16342+C,16343C,16472deletion** 6907R,7573A,8193T,11149G,11653T,12851A,13097A,13489A,15487C,15708C,15787A,15858C,16440C

Polymorphism positions are relative to the sheep reference mitogenome. Polymorphisms in bold font are expected haplotypes for each haplogroup, polymorphisms in regular font are mutations that are not yet associated with identified haplogroups.

# = nucleotide site covered by fewer than 3 reads.

Both membranes of the Waitangi Sheet, as well as the blank piece of parchment, had similar proportions of reads map to the sheep X chromosome and an autosomal chromosome of similar length, indicating that all three parchments were produced from ewes.

Contamination rates were low and comparable to published rates for parchment [3]: 2% for the lower (4 of 200 bases) and upper membranes (3 of 137 bases) of the Waitangi Sheet and 6% (21 of 355 bases) for the blank piece of parchment. The mapDamage2.0 analysis (S1 Fig) indicated that the parchments demonstrated patterns of DNA damage consistent with degraded DNA. The low level of C to T transitions observed is expected given that a uracil intolerant polymerase was used to amplify the libraries.

## Discussion

Our genetic analyses confirm that the blank piece of parchment is almost certainly a missing piece of the lower membrane of the Treaty of Waitangi, Waitangi Sheet. The two parchments shared identical mitogenome sequences, including an unusual heteroplasmic site, and both were made from ewes. This result is unlikely to be the result of contamination or sample mix-up because negative controls were clean, control region sequences generated by Sanger sequencing were identical for independent extracts of the same parchment and the two samples used for constructing the libraries were extracted on different days. Additionally, the sample from the upper membrane of the Waitangi Sheet, which was extracted at the same time as the sample of the lower membrane, had a different mitogenome and the library constructed from the small piece of parchment attached to the Herald Sheet that had few sheep reads provides a further negative control.

The difference in widths between the blank piece of parchment and the lower membrane of the Waitangi Sheet (29 mm) is probably due to shrinkage from either water damage or the water-based treatments, which were undertaken in the early 20^th^ century and the 1970s and 80s. Likewise the change in colour and texture of the parchment could result from the original water damage, treatments, exposure to light or a combination of all three. Such significant change highlights the value of confidently identifying the blank piece of parchment as part of the Waitangi Sheet lower membrane, because it can now be used to aid in the long-term preservation of New Zealand’s most significant document.

Several spurious sequences from our Sanger sequencing suggests that NGS may provide more reliable results than PCR for parchment. Our detection of bovine 16S DNA sequence from the Herald sample 1 through PCR was not repeatable (the sequence was obtained from only one of the three PCRs). However, bovine DNA sequence, in addition to sheep sequence, was detected from multiple independent PCRs from one sample of the upper membrane of the Waitangi Sheet, even though our NGS results indicate the level of bovine contamination in this sample, assessed through the number of reads aligning to the bovine reference mitogenome, was fewer than the other two parchments (Table 2). Additionally, a second sample of this parchment that was extracted independently produced only sheep 16S sequence. Previous studies have also detected multiple sequences derived from different individuals and/or species in single PCRs from parchments [9,10] and bovine DNA has been shown to be a particularly common contaminant of laboratory reagents [28]. We recommend the use of NGS, rather than PCR amplification of mtDNA, for genetic analyses of parchments. As well as providing much greater resolution, the contamination levels appear much lower than PCR based methods.

## Supporting information

S1 Fig Analysis of parchment DNA damage patterns with MapDamage. For each parchment the top panels show the excess of purines (A to G) immediately before reads, which are characteristic for ancient DNA. The lower panels show nucleotide misincorporations for the first and last 25 bases of the mtDNA fragments (5’ C to T misincorporations are shown in red and 3’ G to A misincorporations are shown in blue).
(DOCX)

S1 File Background information on the blank piece of parchment. Relevant information from historic letters regarding the blank piece of parchment is provided. Morphological dimensions of the main Treaty membranes and the blank piece of parchment are also given.
(DOCX)

S1 Table Sheep microsatellite primers and genotyping results. Only the Blank3 sample, which is from the blank piece of parchment, was able to be genotyped.
(DOCX)
