# Supplementary material for: Genetic analysis identifies the missing parchment of New Zealand’s founding document, the Treaty of Waitangi
Source: PLoS One. 2019 Jan 16;14(1):e0210528. doi: 10.1371/journal.pone.0210528 (PMC6334937; doi:10.1371/journal.pone.0210528)
Supplement: S1 Fig — For each parchment the top panels show the excess of purines (A to G) immediately before reads, which are characteristic for ancient DNA. The lower panels show nucleotide misincorporations for the first and last 25 bases of the mtDNA fragments (5’ C to T misincorporations are shown in red and 3’ G to A misincorporations are shown in blue). (DOCX) [file pone.0210528.s001.docx]

**S1 Fig.** **Analysis of parchment DNA damage patterns with MapDamage.** For each parchment the top panels show the excess of purines (A to G) immediately before reads, which are characteristic for ancient DNA. The lower panels show nucleotide misincorporations for the first and last 25 bases of the mtDNA fragments (5’ C to T misincorporations are shown in red and 3’ G to A misincorporations are shown in blue).

Treaty of Waitangi, Waitangi Sheet upper membrane

Treaty of Waitangi, Waitangi Sheet lower membrane

Blank piece of parchment
