# Supplementary material for: Genetic analysis identifies the missing parchment of New Zealand’s founding document, the Treaty of Waitangi
Source: PLoS One. 2019 Jan 16;14(1):e0210528. doi: 10.1371/journal.pone.0210528 (PMC6334937; doi:10.1371/journal.pone.0210528)
Supplement: S1 File — Relevant information from historic letters regarding the blank piece of parchment is provided. Morphological dimensions of the main Treaty membranes and the blank piece of parchment are also given. (DOCX) [file pone.0210528.s002.docx]

**Background information on the blank piece of parchment**

Following the donation of the WBD Mantell Papers to the Alexander Turnbull Library in 1929 the Librarian wrote to the Under Secretary of the Internal Affairs Department, Wellington on the 21^st^ March 1929 as follows:

“Treaty of Waitangi. Amongst some old papers etc. just received for the library was a blue envelope, enclosed, containing a blank sheet of parchment said to be part of the Treaty of Waitangi. It was evidently kept as a relic; but as such relics are of no intrinsic or other value, seeing that any odd bit of parchment could be said to be what this purports to be and no one could prove or disprove it, I send this on to you. If the cut fits, it may be part of the original parchment; and if so its proper place is with that parchment; or if you do not consider it worth keeping, the waste paper basket might receive it.”

This information was passed to Dr Scholefield, Dominion Archivist, General Assembly Library on 25^th^ March from the Under Secretary with the comment “Would you kindly let me know whether you consider it worth keeping”. Another memorandum is sent to Dr Scholefield on the 24^th^ April asking for a response to the first memorandum. On 27^th^ April Dr Scholefield replies:

“Replying to your memorandum of March 25^th^, I have compared the piece of parchment enclosed therewith with the original sheets of the Treaty of Waitangi, and am of opinion that the statement is correct. The envelope in which the parchment reached me is inscribed ‘1865, Treaty of Waitangi Blank Portion of the Original Skin’, and in another place ‘Blank Parchment from the Original and First Part of the Treaty of Waitangi W.B.D.M. M.N.A (?Minister of Native Affairs) 1865’. Mr Mantell was Minister of Native Affairs at that time.

The first sheet of the Treaty of Waitangi consists of two skins laced together. The second skin is not the usual length, and on measuring it I found that the piece in question exactly made up the size of the other skin. The original sheet was badly damaged by rats, and little of the bottom half remains. It is, however, quite possible to discern the lead pencil ruling, and to see that it corresponds exactly with the ruling on the blank portion of skin. There is, therefore, I think no doubt that the blank parchment was cut off the original sheet by Mr Mantell.

I agree with the Librarian of the Alexander Turnbull library that the relic is of no intrinsic value. Nevertheless, as there is no reason to doubt its genuineness, and as it is not worthwhile restoring it to its proper place by binding it to the original sheet, I would suggest that it might be disposed of by presenting the whole or portions of it to such institutions as the Auckland Public Library, the Hocken Library, the Canterbury Public Library and the Libraries of the University Colleges. The parchment is returned herewith”, signed Dr Scholefield, Controller of Dominion Archives.

The last correspondence is from the Under Secretary to Dr Scholefield, Controller of Dominion Archives, General Assembly Library, Parliament Buildings, Wellington, on the 23^rd^ May, as follows:

“Treaty of Waitangi. With reference to previous memoranda regarding the piece of parchment believed to be a blank portion of the original skin containing the Treaty of Waitangi, I have now to state that as the piece of parchment appears to have nothing more than a sentimental value, I have placed it with the original Treaty.”

The copy of this last letter also contains a note to a Mr Kelleher: “Will you please have this piece of parchment and covering envelope placed with the original Treaty?”, and a reply back to Mr Newton (the Under Secretary): “Placed in special envelope suitably endorsed and placed in tin box containing original Treaty” dated 28^th^ May 1929.

These letters are today stored in an Archives file, ref. IA1 3001, 158/67, R19967609.

**Morphological measurements of the parchments**

Treaty of Waitangi, Waitangi Sheet dimensions:

Total 1168 mm (h) X 609 mm (w)

Membrane 1 (M1) (upper sheet) 723 x 609mm

Membrane 2 (M2) (lower sheet) 466 x 600mm

- The repaired document is not square; there is a variance of up to 11mm widthways and 10mm height wise.
- There is an overlap between the two membranes of up to 25mm.
- Inside measurements below are for the individual membranes.

609mm

| 600mm  **723mm**  **722-3mm**  **1168mm**  **1158mm** |
| --- |
| 457mm  466mm |

598mm

The central line is a double line (centre double line or CDL), the others are vertical, single lines (on Membrane 1). The CDL appears to cross from Membrane 1 to Membrane 2.

Treaty of Waitangi, Herald Sheet dimensions:

650mm (H) x 535mm (W) inclusive of repairs, max size, irregular shaped.

Blank piece of parchment dimensions:

Total 259mm (h) X 627mm (w)

627mm

291mm

335mm

CDL

623mm

235mm

259mm

CDL is 1.5mm wide

567mm

The blank parchment has six columns of ruled lines with 7 vertical lines. The centre line is a double ruled line, which is the same as on the Waitangi Sheet but the lines do not line up.

Comparison of the Treaty of Waitangi, Waitangi Sheet lower membrane 2 (lower sheet) with the blank piece of parchment

- The width of the lower edge of Membrane 2 is 598mm but the upper edge of the blank piece of parchment is 627mm wide so there is a difference of 29mm.
- When the piece of parchment’s CDL is lined up with the CDL on Membrane 2, then the piece of parchment’s width is short on left side by 5mm and overlaps on right side by 33mm.
- On placing the piece of parchment’s CDL slightly to the left (where there is a faint impression of a single pencil line, which is a single line on Membrane 2 but double/triple on Membrane1) then the piece of parchment is wider on both sides and the other ruled lines do not line up (i.e. they are closer together). The piece of parchment overlaps on the left by 11mm and on the right by 7mm.
- The shape of the cut edges may match up together, however the lower edge of Membrane 2 is slightly curled under, but the left side of Membrane 2 and the piece of parchment do line up and so do the two column lines on the far left.
