# Supplementary material for: Genetic analysis identifies the missing parchment of New Zealand’s founding document, the Treaty of Waitangi
Source: PLoS One. 2019 Jan 16;14(1):e0210528. doi: 10.1371/journal.pone.0210528 (PMC6334937; doi:10.1371/journal.pone.0210528)
Supplement: S1 Table — Only the Blank3 sample, which is from the blank piece of parchment, was able to be genotyped. (DOCX) [file pone.0210528.s003.docx]

**S1 Table. Sheep microsatellite primers and genotyping results.** Only the Blank3 sample, which is from the blank piece of parchment, was able to be genotyped.

| Primer pair | Reference | Chromosome position | Blank3 alleles  (bp) |
| --- | --- | --- | --- |
| MCM512F  MCM512R | [1] | 2 | n/a |
| MCM53F  MCM53R | [2] | 6 | n/a |
| OarCP20F  OarCP20R | [3] | 21 | 106/103 |
| BM8125F  BM8125R | [4] | 17 | n/a |
| MCMA10F  MCMA10R | [5] | 9 | 121/121 |
| OarFCB20F  OarFCB20R | [6] | 2 | 131/120 |
| OarCP34F  OarCP34R | [7] | 3 | 132/132 |

n/a = no amplification.

**References**

[1] Hulme DJ, Smith AJ, Silk JP, Redwin JM, Beh KJ. Polymorphic sheep microsatellites at the McM2, McM131, McM135, McM136, McM140, McM200, McM214, McM373, McM505, McM507 and McM512 loci. Anim. Genet. 1995; 26: 369-370.

[2] Smith AJ, Hulme DJ, Silk JP, Redwin JM, Beh KJ. Thirteen polymorphic ovine microsatellites. Anim. Genet. 1995; 26: 277–278.

[3] Ede AJ, Pierson CA, Crawford AM. Ovine microsatellites at the OarCP9, OarCP16, OarCP20, OarCP21, OarCP23 and OarCP26 loci. Anim. Genet. 1994; 26: 129-130.

[4] Bishop MD, Kappes SM, Keele JW, Stone RT, Sunden SL et al. A genetic linkage map for cattle. Genetics. 1994;136: 619-639.

[5] Beh KJ, Riffkin CD, Davies KP, di Lenno KL, Maddox JF. 2010 Dinucleotide repeat polymorphism at the ovine McMA7, McMA10, McMA13, McMA16, McMA17, McMA27, McMA29, McMA42, McMA47 and McMA49 loci. Anim. Genet. 2010; 31: 228-229.

[6] Buchanan FC, Galloway SM, Crawford AM. 1994 Ovine microsatellites at the OarFCB5, Oar FCB19, OarFCB20, OarFCB48, OarFCB129 and OarFCB226 loci. Anim Genet. 1994; 25: 60.

[7] Ede AJ, Pierson CA, Crawford AM. Ovine microsatellites at the OarCP34, OarCP38, OarCP43, OarCP49, OarCP73 and OarCP79 loci. Anim. Genet. 1994; 26: 130-131.
